# Convolutional neural networks and mixture of experts for intrusion detection in 5G networks and beyond

**DOI:** 10.3389/frai.2025.1708953

**Published:** 2026-01-05

**Authors:** Loukas Ilias, George Doukas, Vangelis Lamprou, Christos Ntanos, Dimitris Askounis

**Affiliations:** Decision Support Systems Laboratory, School of Electrical and Computer Engineering, National Technical University of Athens, Athens, Greece

**Keywords:** 5G/6G networks, intrusion detection, deep learning, convolutional neural networks, mixture of experts

## Abstract

The advent of 6G/NextG networks offers numerous benefits, including extreme capacity, reliability, and efficiency. To mitigate emerging security threats, 6G/NextG networks incorporate advanced artificial intelligence algorithms. However, existing studies on intrusion detection predominantly rely on deep neural networks with static components that are not conditionally dependent on the input, thereby limiting their representational power and efficiency. To address these issues, we present the first study to integrate a Mixture of Experts (MoE) architecture for the identification of malicious traffic. Specifically, we use network traffic data and convert the 1D feature array into a 2D matrix. Next, we pass this matrix through a convolutional neural network (CNN) layer, followed by batch normalization and max pooling layers. Subsequently, a sparsely gated MoE layer is used. This layer consists of a set of expert networks (dense layers) and a router that assigns weights to each expert's output. Sparsity is achieved by selecting only the most relevant experts from the full set. Finally, we conduct a series of ablation experiments to demonstrate the effectiveness of our proposed model. Experiments are conducted on the 5G-NIDD dataset, a network intrusion detection dataset generated from a real 5G test network, and the NANCY dataset, which includes cyberattacks from the O-RAN 5G Testbed Dataset. The results show that our introduced approach achieves accuracies of up to 99.96% and 79.59% on the 5G-NIDD and NANCY datasets, respectively. The findings also show that our proposed model offers multiple advantages over state-of-the-art approaches.

## 1 Introduction

Fifth-generation (5G) networks have found applications in several domains, including autonomous vehicles, smart factories, smart cities, and healthcare, due to their significant improvements in latency, throughput, and bandwidth (Saad et al., 2020). Although the potential of 5G networks has not been fully investigated, both academia and industry have shifted their attention to 6G networks (Siriwardhana et al., 2021). At the same time, several projects have been funded under the Smart Networks and Services Joint Undertaking (SNS JU)^1^ and the Horizon 2020 programme (Jiang et al., 2021) to facilitate the transition to 6G networks. As new requirements arise in the context of 6G networks, including latency, mobility, peak data rate, spectrum efficiency, area traffic capacity, and network energy efficiency, it is worth noting that new attackers with advanced characteristics also emerge (Porambage et al., 2021). Specifically, advanced security threats, including eavesdropping (Rif‘a-Pous et al., 2024) and jamming (Priyadarshani et al., 2025; Lohan et al., 2024), necessitate the development of intelligent threat mitigation systems. In this context, artificial intelligence (AI) can play a pivotal role in protecting 6G networks against such attacks, thereby enabling the creation of robust systems.

Existing studies train shallow machine learning classifiers, resulting in suboptimal performance and poor generalization. Recently, existing studies have shifted their focus to converting network traffic or packet-level data into images and training convolutional neural networks (CNNs), pretrained CNNs (AlexNet, VGG19, and ResNet), and RNNs (LSTMs and BiLSTMs), and then employing fully connected layers for classification. However, these approaches rely on dense layers doing everything. Network parameters are fixed during training, while inference is performed statically, which demands additional computational resources and increases both training and inference time. On the contrary, the literature review suggests that models conditioned on the input (Han et al., 2022) offer a range of benefits, including efficiency, expressive power, adaptiveness, and compatibility. This is because these models selectively activate their components. Mixture of Experts belongs to the category of input-conditional computation models. Specifically, MoE was originally proposed in Jacobs et al. (1991) and has since found applications across a range of domains (Aljundi et al., 2017; Cai et al., 2024; Shazeer et al., 2017).

To address the aforementioned limitations, we present the first study to integrate MoE layers into a deep neural network for intrusion detection in 5G networks. Specifically, we use network traffic data represented as a feature set, i.e., a 1D array. We reshape this array into a matrix, which is then fed into the CNN, max-pooling, and batch normalization layers. After this, we use a sparsely gated MoE layer (Shazeer et al., 2017), which applies different subsets of layers (experts) and activates only a selected subset of experts, i.e., the *k* most relevant ones, during each forward pass. Experiments are performed on two publicly available datasets, namely the 5G-NIDD dataset (Samarakoon et al., 2022) and the O-RAN 5G Testbed NANCY dataset (Liatifis et al., 2024). Results demonstrate that the proposed method achieves notable benefits over state-of-the-art approaches.

Our main contributions can be summarized as follows:

- To the best of our knowledge, this is the first study employing sparse MoE layers in the intrusion detection task.
- We perform our experiments on two publicly available datasets related to 5G networks.
- We conduct a series of ablation studies to assess the effectiveness of the proposed architecture.

The rest of this article is organized as follows: Section 2 presents existing studies on intrusion detection. Section 3 presents the dataset used for conducting our experiments. Section 4 presents the proposed methodology. Section 5 presents the experimental setup, results, and the ablation experiments. Finally, Section 6 presents some concluding remarks, limitations, and ideas for future work.

## 2 Related research

### 2.1 Traditional machine learning algorithms

Kasongo and Sun (2020) trained traditional machine learning algorithms using the UNSW-NB15 dataset. The authors employed XGBoost to select the most important features, then trained Artificial Neural Networks (ANNs), k-Nearest Neighbors (k-NN), Decision Trees (DT), Logistic Regression, and Support Vector Machine (SVM). The authors stated that ANN achieved the highest performance.

In Thakkar and Lohiya (2023), the authors introduced an approach based on feature selection. Specifically, the introduced feature selection strategy is based on the fusion of statistical importance measures, namely the standard deviation and the difference between the mean and median. The authors performed their experiments on the NSL-KDD, UNSW-NB15, and CICIDS2017 datasets. The authors compared their introduced feature selection approach with existing approaches, including recursive feature elimination, chi-square, correlation-based feature selection, genetic algorithm, mutual information, Relief-f, and Random Forest. Results showed that the proposed approach outperformed existing approaches across all evaluation metrics and datasets.

Mohale and Obagbuwa (2025) used the UNSW-NB15 dataset and trained multiple ML models, including Decision Trees, Multilayer Perceptron (MLP), XGBoost, Random Forest, CatBoost, Logistic Regression and Gaussian Naive Bayes. Next, the authors employed explainable AI algorithms, including LIME, SHAP, and ELI5, to gain insights into feature importance.

### 2.2 Deep neural networks—CNNs and RNNs

Hadi et al. (2024) presented a multi-tier fusion approach in which several models, including CNNs, GANs, and MLPs, were trained. Fusion methods, including minimum, maximum, median, sum, and weighted sum, were employed to combine the outputs of the aforementioned deep learning models. Experiments were conducted on three datasets, including 5G-NIDD, and showed promising results.

Farzaneh et al. (2024) introduced three transfer learning strategies for detecting DoS attacks. Specifically, the authors utilized a source and a target dataset. Regarding the source dataset, the authors used the dataset introduced in Khan et al. (2023), which consists of eight types of DDoS attacks. Regarding the target dataset, the authors utilized the 5G-NIDD dataset. The authors employed CNNs, ResNet, Inception, and BiLSTM. Transfer learning strategies, including freezing some layers and removing the last layer, were employed. Findings showed that the BiLSTM model achieved the best evaluation results.

Sadhwani et al. (2024) used the 5G-NIDD dataset to conduct their experiments. The authors employed variance and correlation reduction, followed by a filter-based feature selection approach using F1 score, to reduce the dimensionality of the input feature set while retaining the most informative features. The authors trained and tested a series of shallow machine learning classifiers, including kNN, Naive Bayes, DT, and Random Forest (RF), as well as deep learning models, including MLP, CNN, LSTM, and CNN-LSTM. Results showed that CNN-LSTM yielded the highest performance.

A different approach was introduced by Djaidja et al. (2024), which focused on the sequential nature of packets in a network flow. A set of features corresponding to header data was extracted. Next, the authors trained a deep learning model consisting of an LSTM (or GRU) layer followed by an attention mechanism. Experiments were conducted on CICIDS2017 and 5G-NIDD datasets. Results showed that GRU coupled with an attention layer achieved the highest performance on the CICIDS2017 dataset, while LSTM with an attention layer achieved the best performance on the 5G-NIDD dataset.

In Lilhore et al. (2024), experiments were performed on CICIDS2017/2018 and UNSW-NB15 datasets. After applying preprocessing techniques and selecting the most relevant features by employing a decision tree classifier in combination with the Mahalanobis distance-based oversampling method, the authors converted the data into images. Their proposed approach includes a MobileNet in conjunction with an SVM classifier. As baselines, the authors used VGG-16, VGG-19, EfficientNet, and Inception-Net. Results showed the strength of the proposed methodology.

A different approach was introduced by Elsayed et al. (2020), which removed socket features and proposed an RNN-based autoencoder. Experiments were conducted on the CICDDoS2019 dataset (Sharafaldin et al., 2019). Results demonstrated the effectiveness of the proposed method.

Agrafiotis et al. (2023) presented a toolkit for converting packets into images. LSTM autoencoders were trained to generate embeddings, followed by a fully connected layer for classification. Experiments on 5G-NIDD demonstrated that the model achieved promising results.

An image-based method, namely MAGNETO, was introduced by Andresini et al. (2021). After transforming the data into images, the authors trained Generative Adversarial Networks (GANs) to generate new images, thereby augmenting the training set. Finally, CNNs were trained on four datasets, namely KDDCUP99,^2^ UNSW-NB15, CICDS2017, and AAGM17.^3^

Computer vision approaches were also introduced by Paolini et al. (2024). Specifically, the authors presented a method used directly at the packet level. A set of features was extracted per packet. After designing the 2D matrix, the authors employed deep learning computer vision models, including Inception, Xception, EfficientNet, MobileNet, DenseNet, ResNet, and a customized CNN. Results showed that the customized CNN yielded the highest results.

Zhang et al. (2020) introduced a method based on SMOTE and Gaussian Mixture Models for addressing data imbalance. Finally, the authors trained a deep learning model consisting of CNN layers. They compared their method with five class-imbalanced processing techniques, including ROS, SMOTE, ADASYN, replacing GMM with RUS, and k-means. The results showed that the proposed approach outperformed existing approaches.

Almuhanna and Dardouri (2025) used a dataset comprising over 5.6 million network traffic records. To address class imbalance, the authors employed the Synthetic Minority Over-sampling Technique (SMOTE). Finally, a weighted soft-voting ensemble strategy was used. Specifically, predictions from XGBoost, Random Forest, Graph Neural Network (GNN), LSTM, and Autoencoder were integrated.

### 2.3 Unsupervised learning

A contrastive learning approach was proposed by Yuan et al. (2024). Specifically, the authors used the 5G-NIDD dataset and an IoT dataset (Mirsky et al., 2018). Next, the authors used both statistical and original packet features (IP, TCP, UDP, and payload). Afterward, the authors employed an unsupervised method using contrastive autoencoders. An innovative loss was proposed that integrates both reconstruction and contrastive losses. Results showed that the proposed approach yielded results similar to those of existing approaches.

An unsupervised learning approach was introduced by Paolini et al. (2023). Specifically, the authors used an autoencoder and passed the latent representation vector through a Gaussian Mixture Model. The authors performed their experiments on the CICIDS2017 dataset. The results showed the strength of the proposed approach.

The study in Binbusayyis and Vaiyapuri (2021) proposed an approach that combines a one-dimensional convolutional autoencoder with a one-class support vector machine. Experiments were performed on the NSL-KDD and UNSW-NB15 datasets. Findings showed the potential of the proposed approach for designing an effective intrusion detection system.

### 2.4 Related research review findings

Existing studies rely on training traditional machine learning classifiers, resulting in suboptimal performance. Image-based methods are employed in conjunction with the development of customized CNNs, pretrained CNNs in the vision domain (AlexNet, Inception, VGG16), and RNNs. However, these methods are fixed during training, perform inference in a static manner, demanding significant computational resources in this way while also increasing training and inference times.

Our study differs from existing research initiatives because we present the first study using sparsely gated MoE layers, in which only a subset of experts is activated during each forward pass. Additionally, instead of aggregating the outputs from all experts, sparsely gated MoE layers keep only the *k* relevant experts.

## 3 Datasets

### 3.1 5G-NIDD

We use the 5G-NIDD dataset to conduct our experiments (Samarakoon et al., 2022). This dataset has been collected using the 5G Test Network (5GTN).^4^ It contains both PCAP files and network traffic data. Unlike existing datasets, benign traffic has been generated using real mobile devices attached to the 5GTN. The advantage of this dataset over existing ones is that it contains features from 5G network flows. Existing datasets, including UNSW-NB15 (Moustafa and Slay, 2015) and CICIDS2017/2018 (Panigrahi and Borah, 2018; Sharafaldin et al., 2018), have limitations, as they were collected in the past in the absence of technological advances. Moreover, the UNSW-NB15 dataset uses a synthetic environment to generate attacks.

Data from 5G-NIDD are extracted from two base stations. The authors in Samarakoon et al. (2022) have made available a *csv* file corresponding to the combined network flow dataset. The 5G-NIDD dataset contains two attack categories: DoS/DDoS and Port Scan. Regarding DoS/DDoS attacks, the following types are included: ICMP flood, UDP flood, SYN flood, HTTP flood, and Slow Rate DoS. Regarding Port Scan attacks, the following types are included: SYN Scan, TCP Connect Scan, and UDP Scan.

#### 3.1.1 Handling missing values

Several methods have been used to impute missing values. For instance, the study in Sadhwani et al. (2024) drops some columns and imputes missing values using column means. However, dropping columns results in a loss of information. In Singh et al. (2024), the authors impute missing data using the median or zero.

In this study, missing values in numerical features are imputed using the class-wise mean, while categorical features are imputed with the most frequent value per class. The number of samples per traffic class is reported in Table 1.

**Table 1:** Samples per traffic class (5G-NIDD).

**Category**	**Number of instances**
Benign	477,737
UDPFlood	457,340
HTTPFlood	140,812
SlowrateDoS	73,124
TCPConnectScan	20,052
SYNScan	20,043
UDPScan	15,906
SYNFlood	9,721
ICMPFlood	1,155

#### 3.1.2 Designing the feature set

In Appendix Table 8, we describe the features used in our experiments. To normalize numerical features, Min-max Scaling is applied, ensuring values are between 0 and 1. Categorical features are one-hot encoded, excluding the first level to prevent redundancy.

In total, we use 78 features. This 1D array is transformed into a 2D matrix, i.e., 6 × 13, and used as input to the CNN layers, which are described in detail in the next section.

### 3.2 NANCY

The NANCY dataset is newly collected. To construct this dataset, a malicious user carries out cyberattacks against various services running on the main operator and the micro-operator. This dataset contains the following attack types: reconnaissance attack, TCP scan, SYN scan, SYN Flood, HTTP Flood, and slow-rate DoS. The authors used CICFlowMeter^5^ to collect network flow data. Due to data imbalance issues, we removed the Slowrate DoS and Reconnaissance Attack from our dataset. Specifically, the NANCY dataset includes 3,553 samples corresponding to Slowrate DoS and 2,044 samples corresponding to Reconnaissance Attack. The number of samples per traffic class is reported in Table 2.

**Table 2:** Samples per traffic class (NANCY Dataset).

**Class**	**Samples**
SYN flood	162,251
TCP scan	133,569
SYN scan	133,164
HTTP flood	87,531
Slowrate DoS	3,553
Reconnaissance attack	2,044
Benign traffic	65,621

Unlike the 5G-NIDD dataset, the NANCY dataset contains no missing values.

#### 3.2.1 Designing the feature set

We keep only the numerical features. The complete feature set is reported in Appendix Table 9. In total, we use 72 features. Min-Max scaling is applied to ensure values fall between 0 and 1. This 1D array is transformed into a 2D matrix, i.e., 6 × 12, and is given as input to CNN layers, which are described in detail in Section 4.

## 4 Methodology

**Figure 1:**
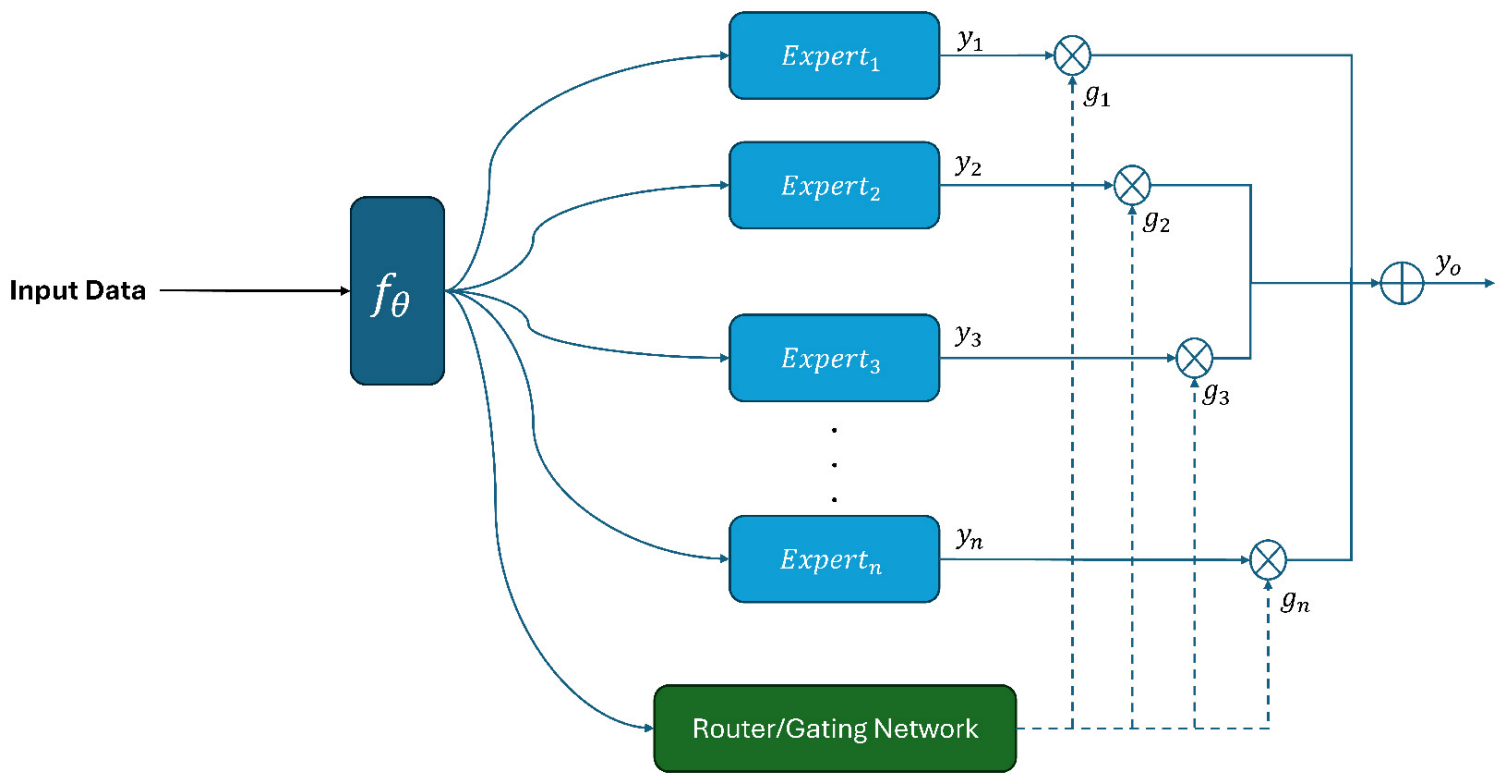
Proposed methodology.

**Figure 2:**
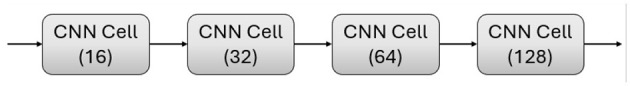
CNN architecture (*f*_θ_).

**Figure 3:**
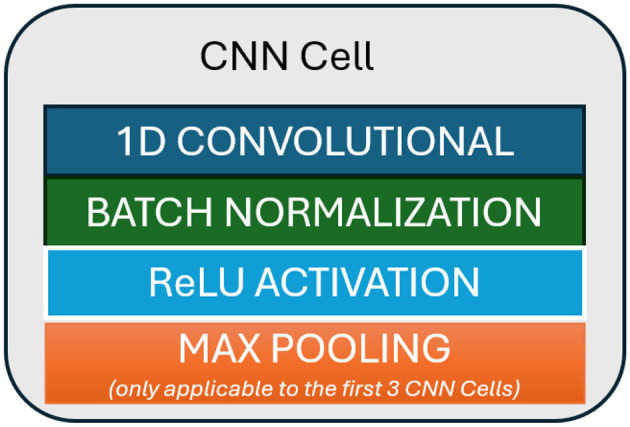
CNN cell structure (see Figure 2).

Our proposed architecture is illustrated in Figures 1–3. Below, we explain in detail each component of our proposed architecture.

### 4.1 Convolutional neural networks

We design a convolutional neural network architecture to obtain a representation vector from input *X*. The CNN component of our methodology is illustrated in Figure 2.

The structure of the CNN cell is illustrated in Figure 3. We place four CNN cells in a row with 16, 32, 64, and 128 filters, respectively. The kernel size and padding are set equal to 3 and 1, respectively. For the max pooling layer, we set both the kernel size and stride to 2. Each CNN cell, except for the final one, consists of a 1D convolution, batch normalization, ReLU activation, and a max-pooling layer. The final CNN cell does not include a max-pooling layer.

Let the output of this model's component be $x=f_{\theta }\left(X\right)\in {\mathbb{R}}^{d}$, where *d* = 128.

### 4.2 Sparse mixture of experts

In this section, we describe the sparsely gated MoE layer. Specifically, this layer consists of *n* experts and a router/gating network. The aim of the router is to determine the important experts based on the input data.

Equation 1 shows the output of the MoE layer, denoted as *y*_*o*_. *y*_*i*_ represents the output of each expert, while *g*_*i*_ indicates the weight assigned by the router to each expert's output *y*_*i*_. Each expert is a deep neural network with a hidden layer of 16 units.


$$\begin{array}{l} y_{o}=\overset{n}{\underset{i=1}{\sum }}g_{i}\left(x\right)y_{i}\left(x\right) \end{array}$$ (1)

Equations 2–4 describe the sparsity mechanism and the addition of noise. Specifically, the *TopK*(·, *k*) function selects the *k* most relevant experts by setting the output vectors to their original values and setting all the other values to −∞. Values of −∞ become close to zero after applying a *softmax* function. The addition of a noise term, denoted as *R*_*noise*_, facilitates load balancing among experts (see below *L*_*load*_) and enhances the stability of MoE training.


$$\begin{array}{l} G{\left(\text{x};\Theta \right)}_{i}=\text{softmax}{\left(\text{TopK}\left(g\left(\text{x};\Theta \right)+R_{\text{noise}},k\right)\right)}_{i}, \end{array}$$ (2)

where ${ℛ}_{\text{noise}}=StandardNormal()\cdot Softplus({\left(x\cdot W_{noise}\right)}_{i})$


$$\text{TopK}{\left(g(x;\Theta ),k\right)}_{i}=\{\begin{array}{ll} g{\left(x;\Theta \right)}_{i}, & \text{condition,}\\ -\infty , & \text{otherwise.} \end{array}$$ (3)

where


$$\begin{array}{l} \text{condition : if }g{\left(\text{x};\Theta \right)}_{i}\text{ is in the top-}k\text{elements of }g\left(\text{x};\Theta \right). \end{array}$$ (4)

For load-balancing purposes and to balance expert utilization, we design two losses based on the study in Shazeer et al. (2017).

- ${ℒ}_{importance}$: Shazeer et al. (2017) observe that large weights are assigned to specific experts by the router. To address this issue and ensure uniform routing weights across all experts, the authors in Shazeer et al. (2017) design the following loss function. This loss aims to assign equal importance to all experts.

$$\begin{array}{l} L_{\text{importance}}=w_{importance}\cdot CV{\left(Importance\left(X\right)\right)}^{2}, \end{array}$$ (5)

where *X* is the batch of features, $CV\left(\cdot \right)=\frac{Std\left(\cdot \right)}{Mean\left(\cdot \right)}$, and *Importance*(·) is defined by the equation below:

$$\begin{array}{l} Importance\left(X\right)=\underset{x\in X}{\sum }G\left(x\right) \end{array}$$ (6)

- ${ℒ}_{load}$: Shazeer et al. (2017) designs the following loss function to ensure that all experts receive an equal number of training instances.

$$\begin{array}{l} P\left(x,i\right)=\Phi \left(\frac{{\left(x\cdot W_{g}\right)}_{i}-kth\text{\_}excluding\left(H\left(x\right),k,i\right)}{Softplus\left({\left(x\cdot W_{noise}\right)}_{i}\right)}\right), \end{array}$$ (7)

where Φ is the cumulative distribution function of the standard normal distribution.

$$\begin{array}{l} Load{\left(X\right)}_{i}=\underset{x\in X}{\sum }P\left(x,i\right), \end{array}$$ (8)

where *Load*(*X*)_*i*_ is the load of the *i*th expert.

$$\begin{array}{l} L_{load}\left(X\right)=w_{load}\cdot CV{\left(Load\left(X\right)\right)}^{2} \end{array}$$ (9)

### 4.3 Loss function

We minimize the following loss function:


$$\begin{array}{l} L=L_{cross-entropy}+\alpha \cdot \left(L_{importance}+L_{load}\right), \end{array}$$ (10)

where $L_{cross-entropy}$ corresponds to the cross-entropy loss function and α is a hyperparameter denoting the importance assigned to these two loss functions.

## 5 Experiments and results

### 5.1 Baselines

In terms of the 5G-NIDD dataset, we compare our method with the following research studies:

- Embeddings & FC (multi-class) (Agrafiotis et al., 2023): this method trains LSTM autoencoders followed by a fully connected layer.
- FC Sehan (multi-class) (Samarakoon et al., 2022): this method trains a Multilayer Perceptron.
- Customized CNN (*N* = 100) (Paolini et al., 2024): this method extracts a set of features per packet, converts flows into an image, and trains a CNN followed by ReLU-activated fully connected layers.
- CNN-LSTM (multi-class) (Sadhwani et al., 2024): this method trains a deep neural network consisting of CNN, LSTM, and fully connected layers.
- Fusion Multi-Tier DNN (Hadi et al., 2024): this method proposes a multi-tier fusion approach, where multiple deep learning models, including MLPs, CNNs, and GANs, are trained with the final classification obtained through some combination rules, including maximum, minimum, median, sum, and weighted sum.

Since the NANCY dataset is newly collected, no research studies have been conducted on it.

### 5.2 Experimental setup

We use PyTorch (Paszke et al., 2019) for performing our experiments. All experiments are conducted on an NVIDIA A100 80GB PCIe GPU.

#### 5.2.1 5G-NIDD

We use 128 experts (value of *n*). We retain the 32 most relevant experts (with a value of *k*). We set α of Equation 10 equal to 0.1. We use a batch size of 1,024. We split the dataset into training and test sets (60%–40%) using stratified sampling. We train the models for up to 40 epochs. The total number of parameters is equal to 473,225.

#### 5.2.2 NANCY

We use 64 experts (value of *n*). We keep the 32 most relevant experts (with value of *k*). We set α of Equation 10 equal to 0.1. We use a batch size of 256. Moreover, we apply class weights. We divide the dataset into a train and test set (70%–30%) in a stratified manner. We train the models for up to 40 epochs. The total number of parameters is equal to 294,798.

### 5.3 Evaluation metrics

Precision, recall, and F1 score are reported for each class. Accuracy is also included.

Regarding 5G-NIDD, we also report the weighted F1 score given the dataset's imbalance.

### 5.4 Results

#### 5.4.1 Results on the 5G-NIDD dataset

Results of our introduced methodology are reported in Table 3. Specifically, this table reports the results, i.e., precision, recall, and F1 score, per class and the overall accuracy. We observe that our introduced model achieves an F1 score greater than 0.99900 for the Benign, TCP Connect Scan, ICPM flood, and UDP flood classes. Specifically, our model perfectly identifies ICPM flood and UDP flood attacks, achieving a score of 1 across all metrics. For the SYN Scan, SYN flood, and HTTP flood classes, the model yields FI scores greater than or equal to 0.99800 but below 0.99900. Finally, we observe that our model does not perform well at recognizing UDP Scan and Slow Rate DoS attacks, with F1 scores of 0.99796 and 0.99735, respectively.

**Table 3:** Results of our proposed approach (5G-NIDD).

**Class**	**Accuracy**	**Precision**	**Recall**	**F1 Score**
Benign	0.99958	0.99996	0.99993	0.99995
SYN scan		0.99888	0.99713	0.99800
TCP connect scan		0.99975	0.99950	0.99963
UDP scan		0.99702	0.99890	0.99796
ICPM flood		1.0	1.0	1.0
UDP flood		1.0	1.0	1.0
SYN flood		0.99846	0.99949	0.99897
HTTP flood		0.99793	0.99922	0.99857
Slow rate DoS		0.99849	0.99621	0.99735

Table 4 reports the results of our method in comparison with state-of-the-art approaches. As shown, our proposed model outperforms existing studies by 0.00044–0.00875, 0.00043–0.01577, and 0.00148–0.00835 in precision, recall, and accuracy, respectively. Our method also outperforms existing studies in F1 score by 0.01228-0.00194, except for *Customized CNN (N=100) (Paolini et al., 2024)*. However, it must be noted that the study by Paolini et al. (2024) outperforms ours by a small margin of 0.00001. In our study, we also report a weighted F1 score to account for data imbalance. As shown, our proposed model achieves a weighted F1 score of up to 0.99958. Therefore, our study, which considers conditional-input components, offers lower training and inference times, among other benefits, and achieves performance comparable to state-of-the-art results.

**Table 4:** Performance comparison among proposed models and baselines on the 5G-NIDD dataset.

	**Evaluation metrics**				
**Architecture**	**Precision**	**Recall**	**F1 score**	**Accuracy**	**Weighted F1 score**
**Comparison with state-of-the-art**					
Embeddings & FC (multi-class) (Agrafiotis et al., 2023)	0.99019	0.98316	0.98666	0.99123	–
FC Sehan (Samarakoon et al., 2022)	0.99167	0.98869	0.99017	0.99499	–
Customized CNN (*N* = 100) (Paolini et al., 2024)	-	-	0.99895	–	–
CNN-LSTM (multi-class) (Sadhwani et al., 2024)	0.99850	0.99850	0.99700	0.99810	–
Fusion multi-tier DNN (Hadi et al., 2024)	–	–	–	0.99150	–
**Introduced approach**					
	0.99894	0.99893	0.99894	0.99958	0.99958

#### 5.4.2 Results on the NANCY dataset

Table 5 presents the performance metrics of the proposed approach on the NANCY dataset, including accuracy, precision, recall, and F1 score for five traffic classes: Benign, SYN Scan, TCP Connect Scan, SYN flood, and HTTP flood. The overall accuracy of the model across all classes is 0.79592, indicating that approximately 79.6% of instances were correctly classified. Among the individual classes, the benign class achieved high performance, with a precision of 0.99637, a recall of 0.97739, and an F1 score of 0.98679, indicating that normal traffic was accurately detected with minimal misclassifications.

**Table 5:** Results of our proposed approach on the NANCY dataset.

**Class**	**Accuracy**	**Precision**	**Recall**	**F1 score**
Benign	0.79592	0.99637	0.97739	0.98679
SYN scan		0.59807	0.39572	0.47629
TCP connect scan		0.64581	0.74404	0.62969
SYN flood		0.99865	0.98469	0.99162
HTTP flood		0.99787	0.99760	0.99774

In contrast, the model struggled to detect SYN Scan traffic, achieving the lowest performance metrics: precision of 0.59807, recall of 0.39572, and F1 score of 0.47629. This indicates a significant number of false negatives, pointing to challenges in identifying this type of scan-based intrusion. TCP Connect Scan performed moderately well, with a precision of 0.64581, a recall of 0.74404, and an F1 score of 0.62969, indicating a more balanced but still limited detection capability.

On the other hand, the approach showed excellent performance in detecting flooding attacks. SYN flood and HTTP flood traffic were classified with high precision and recall values 0.99865 and 0.98469 for SYN flood, and 0.99787 and 0.99760 for HTTP flood, respectively resulting in F1 scores of 0.99162 and 0.99774. These results demonstrate the model's robustness in identifying high-volume, aggressive attacks while also highlighting the need to improve detection of subtler, low-rate intrusion attempts, such as SYN scans.

#### 5.4.3 Comparative analysis of results

As one can observe, performance on the NANCY dataset is lower than the performance obtained on the 5G-NIDD dataset. We speculate that this difference in performance is attributable to (i) fewer samples overall. 5G-NIDD contains approximately 1.2 million flows, whereas NANCY provides around 0.58 million after class pruning, i.e., less than half the data, which limits model capacity utilization and increases variance in the router's estimates; (ii) Dataset/feature shift. 5G-NIDD includes flow features extracted via the Argus tool (78 total, with categorical features one-hot encoded), whereas NANCY is produced by CICFlowMeter (72 numeric features only). This change in feature space and traffic generation process is attributable to the difference in performance; (iii) Attack generation shift. Regarding 5G-NIDD, attacker nodes are Raspberry Pi 4 devices that connect to the 5GTN RAN via Huawei E6878 5G modems (the modem acts as the UE). Their target is an Ubuntu server deployed in the 5G Test Network Multi-access Edge Computing (MEC) environment. For the application-layer scenarios, the MEC host runs an Apache2 web server. On the other hand, NANCY's attacks target services in a 5G coverage-expansion scenario involving a main operator and a micro-operator that extends the main operator's coverage; the testbed includes O-RAN elements (a near-RT RIC over the E2 interface) used to collect RAN metrics.

### 5.5 Ablation study

In this section, we conduct a series of ablation studies to assess the effectiveness of the proposed architecture. The results of the ablation study are shown in Table 6.

**Table 6:** Ablation study (5G-NIDD dataset).

**Architecture**	**Evaluation metrics**			
	**Accuracy**	**Precision**	**Recall**	**F1 score**
**Set importance and load loss to zero**				
Benign	0.99855	0.99996	0.99989	0.99993
SYN scan		0.99925	0.99576	0.99750
TCP connect scan		0.99988	0.99950	0.99969
UDP scan		0.99484	0.99937	0.99710
ICPM flood		1.0	1.0	1.0
UDP flood		1.0	1.0	1.0
SYN flood		0.99846	0.99949	0.99897
HTTP flood		0.99113	0.99741	0.99426
Slow rate DoS		0.99485	0.98311	0.98894
**Remove MoE layer**				
Benign	0.99829	0.99996	0.99987	0.99992
SYN scan		0.99925	0.99538	0.99731
TCP connect scan		0.99975	0.99950	0.99963
UDP scan		0.99452	0.99921	0.99686
ICPM flood		0.99569	1.0	0.99784
UDP flood		1.0	1.0	1.0
SYN flood		0.99795	0.99949	0.99871
HTTP flood		0.98819	0.99837	0.99325
Slow rate DoS		0.99658	0.97726	0.98683
**Simple input and dense layer**				
Benign	0.99156	0.99996	0.98970	0.99480
SYN scan		0.99888	0.99738	0.99813
TCP connect scan		0.99988	0.99776	0.99881
UDP Scan		0.99639	0.99827	0.99733
ICPM flood		0.99355	1.0	0.99676
UDP flood		0.99997	1.0	0.99999
SYN flood		0.97418	0.99949	0.98667
HTTP flood		0.95971	0.98407	0.97174
Slow rate DoS		0.94523	0.95938	0.95225

#### 5.5.1 5G-NIDD

First, we set *L*_*load*_ = *L*_*importance*_ = 0. Results showed a decrease in accuracy of 0.00103. Specifically, the F1 scores for the Benign, SYN Scan, UDP Scan, and HTTP flood decreased by 0.00002, 0.0005, 0.00086, and 0.00431, respectively. By setting both losses to zero, the router allocates more training instances to the relevant experts and assigns higher weights to them. This fact degrades the performance of the MoE layer.

Second, we remove the MoE layer and replace it with a dense layer. To be more specific, the output vector of the CNN layers (128*D*) is passed through a dense layer consisting of nine units (output layer). Results showed that accuracy decreased from 0.99958 to 0.99829. Specifically, the F1 scores for the Benign, SYN Scan, ICPM flood, SYN flood, HTTP flood, and slow-rate DoS decreased by 0.00003, 0.00069, 0.00216, 0.00026, 0.00532, and 0.01052, respectively.

Finally, we remove both the CNN and MoE layers and train a deep neural network with an input layer (78 units) and an output layer (nine units). Findings showed that accuracy decreased by 0.00802. F1 scores for all classes, except SYN Scan, decreased. Specifically, the F1 scores for Benign, TCP Connect Scan, UDP Scan, ICPM flood, UDP flood, SYN flood, HTTP flood, and Slow rate DoS decreased by 0.00515, 0.00082, 0.00063, 0.00324, 0.00001, 0.0123, 0.02683, and 0.0451, respectively.

**Figure 4:**
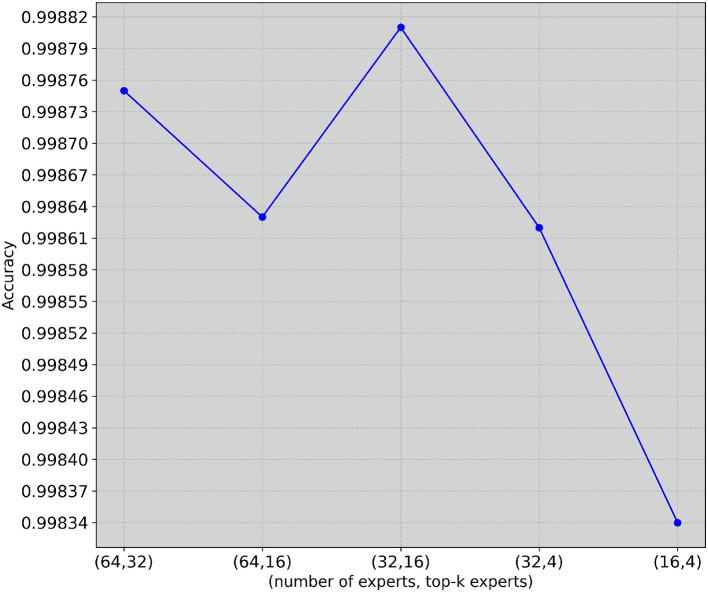
Ablation study (5G-NIDD dataset). Varying the number of experts and top-*k* experts.

Finally, in Figure 4, we vary the number of experts (*n*) and the number of *k* indicating the top experts. On the *x*-axis, (*n, k*) is reported, and *n* corresponds to the number of experts, where *k* corresponds to the number of the most relevant experts as described in Equation 3. On the *y*-axis, accuracy is reported. As one can easily observe, in the majority of cases, reducing the number of experts also reduces accuracy. Specifically, (64, 32) corresponds to an accuracy of 0.99875, (64, 16) corresponds to an accuracy of 0.99863, (32, 16) corresponds to an accuracy of 0.99881, (32, 4) corresponds to an accuracy of 0.99862, and (16, 4) corresponds to an accuracy of 0.99834. Therefore, having more experts leads to better performance for our task.

#### 5.5.2 NANCY

First, we set *L*_*load*_ = *L*_*importance*_ = 0. Our proposed model (Table 5) achieved an overall accuracy of 0.79592, whereas this model (Table 7) shows a slightly lower accuracy of 0.78614. While the difference in overall accuracy is minor, a closer examination of class-wise performance reveals more insightful distinctions. For the Benign class, the first model slightly outperforms the second, with an F1 score of 0.98679 compared to 0.98389, indicating marginally better consistency in identifying normal traffic. In the case of SYN Scan detection, the first model again shows superior performance across all metrics: precision (0.59807 vs. 0.57368), recall (0.39572 vs. 0.35541), and F1 score (0.47629 vs. 0.43890). Although both models struggle to detect SYN scans, the first model demonstrates a better capacity to identify this type of stealthy intrusion. For TCP Scan (Connect Scan), the second model performs almost identically to the first in recall (0.74533 vs. 0.74404) but has slightly lower precision (0.52975 vs. 0.64581) and F1 score (0.61932 vs. 0.62969). This suggests that while both models detect similar quantities of true positives, the first model does so with fewer false positives, making it more reliable overall for this class. Regarding SYN flood detection, the F1 scores are nearly identical (0.99070 vs. 0.99162), indicating both approaches are highly effective for this attack type with negligible differences. Finally, for HTTP flood detection, one observes a drop in the F1 score from 0.99774 to 0.99718.

**Table 7:** Ablation study (NANCY dataset).

**Architecture**	**Evaluation metrics**			
	**Accuracy**	**Precision**	**Recall**	**F1 score**
**Set importance and load loss to zero**				
Benign	0.78614	0.99486	0.97317	0.98389
SYN scan		0.57368	0.35541	0.43890
TCP Scan		0.52975	0.74533	0.61932
SYN flood		0.99754	0.98395	0.99070
HTTP flood		0.99802	0.99635	0.99718
**Remove MoE layer**				
Benign	0.77876	0.99355	0.97815	0.98579
SYN scan		0.51127	0.83337	0.63374
TCP Scan		0.59182	0.23221	0.33354
SYN flood		0.99496	0.98662	0.99077
HTTP flood		0.99817	0.99589	0.99703
**Simple input and dense layer**				
Benign	0.76558	0.97819	0.95929	0.96865
SYN Scan		0.49875	0.80135	0.61484
TCP Scan		0.54528	0.23689	0.33029
SYN flood		0.99721	0.97579	0.98638
HTTP flood		0.98796	0.98394	0.98595

Next, we remove the MoE layer and observe the resulting performance differences. Our proposed model demonstrates better overall accuracy (0.79592 vs. 0.77876). For benign traffic, both models perform similarly, but the proposed model achieves marginally higher precision and an F1 score, indicating slightly more reliable identification of normal activity. In detecting SYN scan attacks, the model without the MoE layer shows a significantly higher recall (0.83337 vs. 0.39572), meaning it captures more true positives, while the model with the MoE layer maintains higher precision, resulting in a better overall balance. For TCP scan, the introduced model clearly outperforms the model without MoE with much higher recall (0.74404 vs. 0.23221) and F1 score (0.62969 vs. 0.33354), highlighting the proposed model's superior ability to detect this attack type. Both models perform exceptionally well at detecting SYN and HTTP floods, with the model without the MoE layer achieving slightly higher recall for SYN flood detection, though the difference is minimal. Overall, our proposed model, which incorporates both the CNN and MoE components, provides a more balanced and consistent performance across all traffic classes.

Finally, we use a neural network with 72 units (input layer), 36 units (hidden layer), and five units (output layer). Results show that CNN+MoE outperforms the dense-layer model in terms of overall accuracy (0.79592 vs. 0.76558), indicating better general classification performance. For benign traffic, CNN+MoE achieves slightly higher precision, recall, and F1 score, suggesting it identifies normal behavior more accurately. In detecting SYN scan attacks, the dense-layer model shows notably better recall (0.80135 vs. 0.39572), detecting more true positives, while CNN + MoE maintains higher precision. For TCP scan detection, both models perform weakly, but CNN + MoE again provides a better balance, with significantly higher recall (0.74404 vs. 0.23689) and F1 score (0.62969 vs. 0.33029). In flood-based attacks, both models perform very well; however, CNN + MoE slightly outperforms the dense-layer model on HTTP flood detection, while both are comparably strong for SYN flood. Overall, CNN+MoE exhibits more consistent and balanced performance across all classes, particularly in scan-type attack detection.

## 6 Conclusion and future research

In this study, we present the first application of the Mixture of Experts to the intrusion detection task. Specifically, we use a publicly available dataset (5G-NIDD) generated from a real 5G test network. An input 1D array of features is transformed into a 2D matrix and then fed into the CNN layers. The representation vector from the CNN layers is passed through the Mixture of Experts layer, which consists of experts and a router. Results on the 5G-NIDD dataset showed that our proposed model outperforms the state of the art, achieving accuracies of 0.99958 and F1 scores of 0.99894. Results on the NANCY dataset indicate that the proposed approach reaches an accuracy of up to 0.78614. An ablation study demonstrated the effectiveness of all the components of our introduced approach.

### 6.1 Limitations

Our study has certain limitations. Specifically, we used simple methods for imputing missing values, which may influence the evaluation performance. More advanced methods, including Generative Adversarial Imputation Networks (GAIN) (Yoon et al., 2018), have been proposed over the years. Additionally, our study relies on labeled datasets.

### 6.2 Future research

In the future, we aim to use advanced missing value imputation methods and combine self-supervised learning with a mixture of expert strategies. Another plan is to use transfer learning strategies (e.g., pretraining on one dataset and fine-tuning on another), assess performance in real-time intrusion-detection settings, and investigate lightweight MoE variants for edge deployment.

## Data Availability

The data analyzed in this study can be found at: 5G-NIDD: https://etsin.fairdata.fi/dataset/9d13ef28-2ca7-44b0-9950-225359afac65; NANCY: https://zenodo.org/records/14811122.

## Footnotes

1
https://smart-networks.europa.eu/

2
https://kdd.ics.uci.edu//databases//kddcup99//kddcup99.html

3
https://www.unb.ca/cic/datasets/android-adware.html

4
https://5gtnf.fi/

5
https://www.unb.ca/cic/research/applications.html

## Appendix

**Table 8:** Description of features.

**Features**	**Description**	**Type**	**No. of features after preprocessing**
Seq	Sequence Number	Numerical	1
Dur	Duration	Numerical	1
RunTime	Run Time	Numerical	1
Mean	Mean Value	Numerical	1
Sum	Sum Value	Numerical	1
Min	Minimum Value	Numerical	1
Max	Maximum Value	Numerical	1
Proto	Protocol Type	Categorical	7
sTos	Type of Service values	Numerical	1
dTos		Numerical	1
sDSb	Source and Destination Behavioral Flags	Categorical	11
dDSb		Categorical	5
sTtl	Time-to-Live values for source and destination	Numerical	1
dTtl		Numerical	1
sHops	Number of hops taken by packets from source to destination	Numerical	1
dHops		Numerical	1
Cause	Cause code	Categorical	2
TotPkts	Total Packets	Numerical	1
SrcPkts	Source Packets	Numerical	1
DstPkts	Destination Packets	Numerical	1
TotBytes	Total Bytes	Numerical	1
SrcBytes	Source Bytes	Numerical	1
DstBytes	Destination Bytes	Numerical	1
Offset	Offset Value	Numerical	1
sMeanPktSz	Source Mean Packet Size	Numerical	1
dMeanPktSz	Destination Mean Packet Size	Numerical	1
Load	Load Value	Numerical	1
SrcLoad	Source Load	Numerical	1
DstLoad	Destination Load	Numerical	1
Loss	Total Loss	Numerical	1
SrcLoss	Source Loss	Numerical	1
DstLoss	Destination Loss	Numerical	1
pLoss	Packet Loss	Numerical	1
SrcGap	Time Gaps	Numerical	1
DstGap		Numerical	1
Rate	Transmission Rates	Numerical	1
SrcRate		Numerical	1
DstRate		Numerical	1
State	State Information	Categorical	10
SrcWin	TCP Window Sizes	Numerical	1
DstWin		Numerical	1
sVid	VLAN IDs	Numerical	1
dVid		Numerical	1
SrcTCPBase	TCP Base Values	Numerical	1
DstTCPBase		Numerical	1
TcpRtt	TCP Round Trip-Time	Numerical	1
SynAck	Specific Packet Types	Numerical	1
AckDat		Numerical	1
**Total**	-	-	**78**

**Table 9:** Description of Features used (NANCY dataset).

**Feature**	**Description**	**Feature**	**Description**
Flow Duration	Duration of the flow in microseconds	Tot Fwd Pkts	Total number of packets in the forward direction
Tot Bwd Pkts	Total number of packets in the backward direction	TotLen Fwd Pkts	Total length of packets in the forward direction
TotLen Bwd Pkts	Total size of packets in the backward direction	Fwd Pkt Len Max	Maximum size of forward packets
Fwd Pkt Len Min	Minimum size of forward packets	Fwd Pkt Len Mean	Mean size of forward packets
Fwd Pkt Len Std	Standard deviation size of packet in forward direction	Bwd Pkt Len Max	Maximum size of packets in backward direction
Bwd Pkt Len Min	Minimum size of backward packets	Bwd Pkt Len Mean	Mean size of backward packets
Bwd Pkt Len Std	Standard deviation size of packet in backward direction	Flow Byts/s	Number of flow bytes per second
Flow Pkts/s	Number of flow packets per second	Flow IAT Mean	Mean time between two packets sent in the flow
Flow IAT Std	Standard deviation time between two packets sent in the flow	Flow IAT Max	Maximum time between two packets sent in the flow
Flow IAT Min	Minimum time between two packets sent in the flow	Fwd IAT Tot	Total time between two packets sent in the forward direction
Fwd IAT Mean	Mean time between two packets sent in the forward direction	Fwd IAT Std	Standard deviation time between two packets sent in the forward direction
Fwd IAT Max	Maximum time between two packets sent in the forward direction	Fwd IAT Min	Minimum time between two packets sent in the forward direction
Bwd IAT Tot	Total time between two packets sent in the backward direction	Bwd IAT Mean	Mean time between two packets sent in the backward direction
Bwd IAT Std	Standard deviation time between two packets sent in the backward direction	Bwd IAT Max	Maximum time between two packets sent in the backward direction
Bwd IAT Min	Minimum time between two packets sent in the backward direction	Fwd Header Len	Total header length in forward direction
Bwd Header Len	Total header length in backward direction	Fwd Pkts/s	Number of forward packets per second
Bwd Pkts/s	Number of backward packets per second	Pkt Len Min	Minimum packet length
Pkt Len Max	Maximum packet length	Pkt Len Mean	Mean packet length
Pkt Len Std	Standard deviation of packet lengths	Pkt Len Var	Variance of packet lengths
FIN Flag Cnt	Number of packets with FIN flag	SYN Flag Cnt	Number of packets with SYN flag
RST Flag Cnt	Number of packets with RST flag	PSH Flag Cnt	Number of packets with PSH flag
ACK Flag Cnt	Number of packets with ACK flag	URG Flag Cnt	Number of packets with URG flag
CWE Flag Count	Number of CWE flag packets	ECE Flag Cnt	Number of packets with ECE flag
Down/Up Ratio	Ratio of bytes sent in backward and forward directions	Pkt Size Avg	Average packet size
Fwd Seg Size Avg	Average segment size in forward direction	Bwd Seg Size Avg	Average segment size in backward direction
Fwd Byts/b Avg	Average number of bytes per bulk in forward direction	Fwd Pkts/b Avg	Average number of packets per bulk in forward direction
Fwd Blk Rate Avg	Average bulk rate in forward direction	Bwd Byts/b Avg	Average bytes per bulk in backward direction
Bwd Pkts/b Avg	Average packets per bulk in backward direction	Bwd Blk Rate Avg	Average bulk rate in backward direction
Subflow Fwd Pkts	Total packets in forward subflows	Subflow Fwd Byts	Total bytes in forward subflows
Subflow Bwd Pkts	Total packets in backward subflows	Subflow Bwd Byts	Total bytes in backward subflows
Init Fwd Win Byts	Initial window bytes in forward direction	Init Bwd Win Byts	Initial window bytes in backward direction
Fwd Act Data Pkts	Number of forward active data packets	Fwd Seg Size Min	Minimum segment size in forward direction
Active Mean	Mean active time	Active Std	Std of active time
Active Max	Maximum activity time	Active Min	Minimum activity time
Idle Mean	Mean idle time	Idle Std	Std of idle time
Idle Max	Maximum idle time	Idle Min	Minimum idle time
